# 

**Published:** 1882-03

**Authors:** 


ESTABLISHED IN 1855.

OLD SERIES. Vol XIX—No. 12. MARCH, 1882. NEW SERIES. Vol. I—No. 6

### ATLANTA MEDICAL REGISTER.

*(ATLANTA MEDICAL AND SURGICAL JOURNAL.)*

S.G.O. INDEXED

EDITORS:

JNO. THAD. JOHNSON, M.D., JAMES B. BAIRD, M.D.

PUBLISHED BY H. H. DICKSON, AT $2.50 A YEAR IN ADVANCE

ATLANTA, GEORGIA:

*H. H. DICKSON, BOOK AND JOB PRINTER AND BOOKBINDER.*

1882.

